# ATM Kinase Small Molecule Inhibitors Prevent Radiation-Induced Apoptosis of Mouse Neurons In Vivo

**DOI:** 10.3390/kinasesphosphatases2030017

**Published:** 2024-09-18

**Authors:** Yüksel Aydar, Sanara S. Rambukkanage, Lauryn Brown, Juan Wang, Ji Sung Seo, Keming Li, Yong Cheng, Laura Biddlestone-Thorpe, Caila Boyd, Amrita Sule, Kristoffer Valerie

**Affiliations:** 1 Department of Anatomy, Medical School of Osmangazi University, Eskisehir 26040, Turkiye;; 2 Massey Comprehensive Cancer Center, Department of Radiation Oncology, Virginia Commonwealth University, Richmond, VA 23298, USA;

**Keywords:** ATMi, central nervous system, DNA damage response, glioma, PP2A, p53

## Abstract

ATM kinase is becoming an important therapeutic target for tumor radiosensitization. Radiation is known to cause neuro-inflammation and neurodegeneration; however, the effects of small molecule ATM inhibitors (ATMi’s) and radiation on normal tissue, including healthy brain, are largely unexplored. Therefore, we examined the mouse CNS after ATMi radiosensitization with a focus on the fate of neurons. We used several approaches to assess the effects on the DNA damage response (DDR) and apoptosis of neurons using immunostaining. In vivo, a significant decrease in viable neurons and increase in degenerating neurons and apoptosis was observed in mice treated with radiation alone. On the other hand, an ATMi alone had little to no effect on neuron viability and did not induce apoptosis. Importantly, the ATMi’s did not further increase radiation toxicity. In fact, multiplex immunostaining showed that a clinical candidate ATMi (AZD1390) protected mouse neurons from apoptosis by 90% at 4 h after radiation. We speculate that the lack of toxicity to neurons is due to a normal ATM–p53 response that, if blocked transiently with an ATMi, is protective. Altogether, in line with previous work using ATM knockout mice, we provide evidence that ATM kinase inhibition using small molecules does not add to neuronal radiation toxicity, and might, in fact, protect them from radiation-induced apoptosis at least in the short term.

## 1. Introduction

ATM kinase plays a critical role in the response to DNA damage and is a pivotal regulator of the cell cycle, DNA damage response (DDR), and other important cellular processes such as inflammation, senescence, insulin resistance, and aging [1–4]. During the past decade, we have explored the basic biological and therapeutic effects of various ATMi’s as radiosensitizers for treating brain cancers such as glioblastoma multiforme (GBM) and demonstrated impressive radiosensitization in preclinical tumor models [5–12]. Our early findings were recently recapitulated by others using patient-derived xenografts [13]. GBM standard-of-care (SOC) is surgical resection followed by chemo-radiation [14]. Radiation of the brain is known to cause neuro-inflammation and neurodegeneration in rodents and humans [15,16]. Therefore, it is of critical importance to determine the effects of ATMi’s and radiation on healthy brains even though today’s glioma SOC uses conformal radiation, considerably reducing the damage to surrounding healthy brain tissue.

Early work on an ATM knockout (KO) mouse demonstrated resistance to apoptosis in the developing mouse central nervous system (CNS) after ionizing radiation that was mediated by p53 in brain regions known to harbor neurons such as the hippocampus and cerebellum [17]. However, the identity of these cells was not conclusively shown to be neurons by immunostaining. Subsequently, the same group found that astrocytes (GFAP+) from the ATM KO mouse did not show such ATM-dependent radioresistance compared with normal astrocytes and that ATM cooperated with Bax, resulting in apoptosis via caspase-3 activation [18,19]. Along the same line, rat neurons cultured in vitro and challenged with various DNA-damaging agents including radiation re-entered the cell cycle via G_0_*→*G_1_*→*S and then died during mitosis that required Cdc25A. Neurons were protected against apoptosis when also treated with caffeine or wortmannin, which are both nonspecific ATM inhibitors [20]. Thus, contrary to aggressive tumors such as GBM, terminally differentiated neurons are not radiosensitized when ATM is inactivated, suggesting that rat neurons also require ATM to die. Based on these studies, one might argue that specific small molecule ATM inhibitors would radiosensitize gliomas and protect neurons, thus being a cancer-specific treatment [5].

Because neurons do not cycle, they are affected differently from brain tumors. It is possible that temporarily preventing radiation-induced neuronal apoptosis with an ATMi could provide protection until the acute toxicity subsides. The question for some time has been whether an ATM small molecule inhibitor would be protective in the shorter term to achieve cancer specificity. We previously demonstrated that the p53 status of gliomas plays a critical role in the response to ATMi-mediated radiosensitization [7–9], and speculated on the possibility of neuronal protection by ATMi’s [5]. If gliomas with mutant/dysregulated p53 signaling respond better to ATMi radiosensitization compared with p53 wildtype tumors as well as normal, healthy brains [5,6], thereby providing significant cancer specificity, then the simultaneous protection of neurons from apoptosis would potentially spare normal brain tissue and perhaps better preserve post-treatment cognition. In addition to the question of whether an ATMi would provide protection to irradiated neurons, it is crucial to keep in mind that prolonged exposure to an ATMi is likely detrimental to dividing cells since blocking the DDR, preventing DNA double-strand break (DSB) repair and other critical cellular processes, would likely negatively affect brain function and result in neurodegeneration and premature aging [21]. The question is whether an ATMi would provide protection to neurons especially if applied after irradiation and, if so, what is the inhibition time window needed post-irradiation to achieve protection?

We set out to determine the impact of ATMi’s on the mouse CNS after irradiation and we found that inhibiting ATM kinase did not adversely affect the survival of neurons in combination with radiation and was in fact protective in the short term.

## 2. Results

### 2.1. Effects of Radiation on Mouse Tumors and Healthy Brains—Radiation Triggers Mitotic Catastrophe in Mouse Tumors

**Figure 1.**
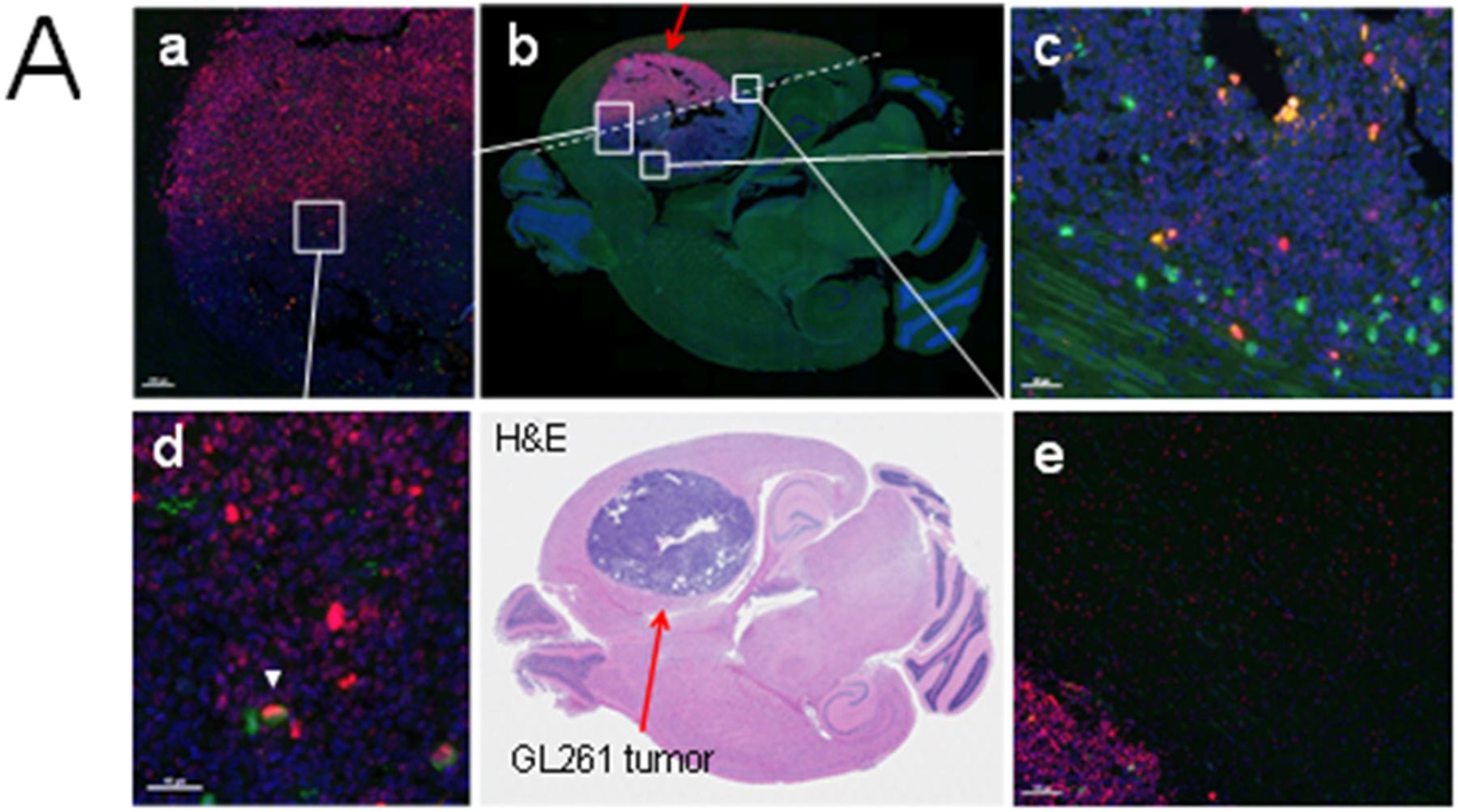

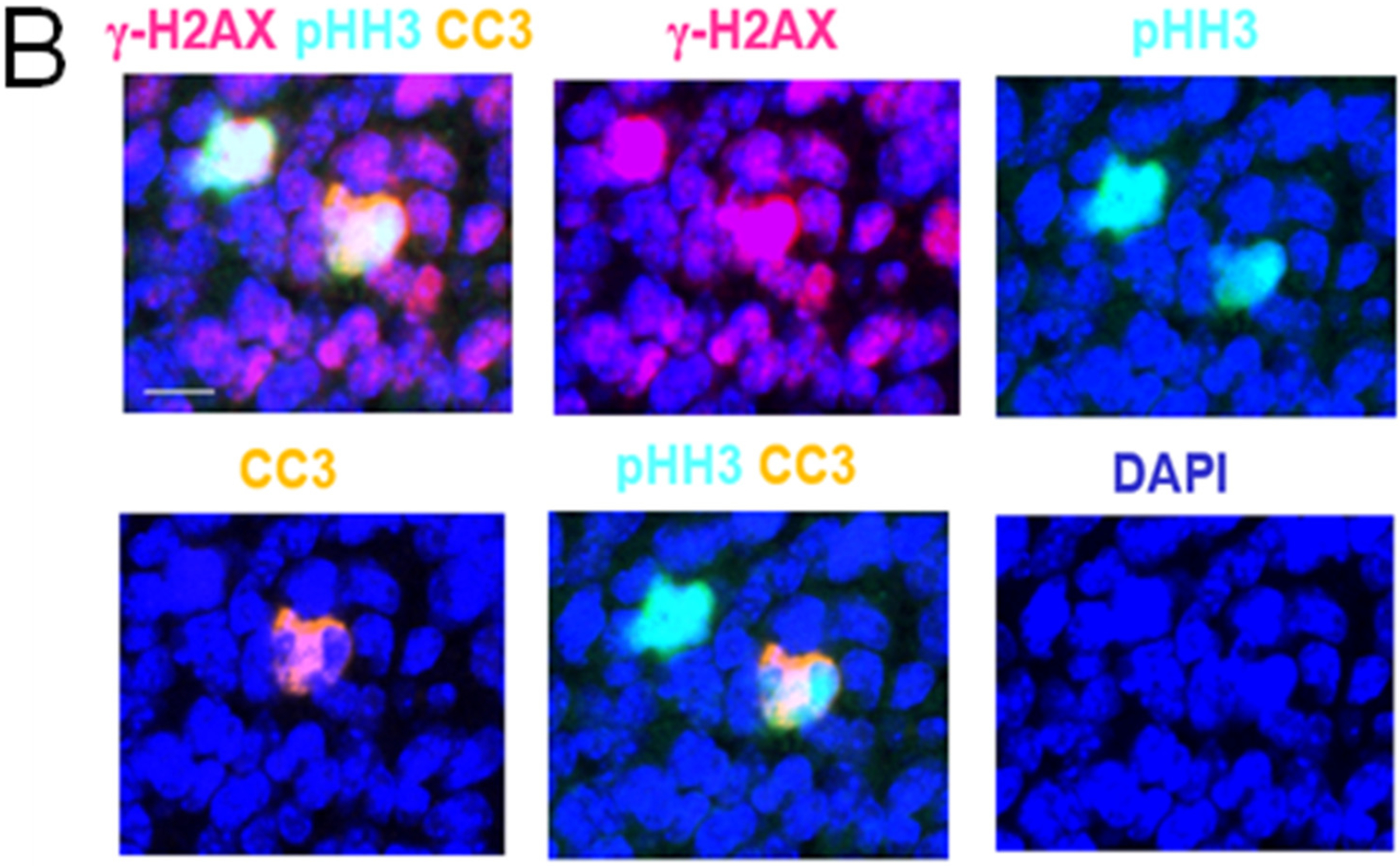
(**A**) Mitotic catastrophe documented by OPAL triple fluorescence staining of brain section with partially irradiated GL261 tumor. A C57bl6 mouse with a GL261 intra-cranial tumor (red arrow, bottom-middle panel) was partially irradiated (5 Gy) using SARRP and a 3 × 9 mm collimator; 1 h later, the brain was fixed and triple stained with anti-γ-H2AX/CC3/pHH3 antibodies and DAPI. DNA-damaged cells were detected in both tumor and healthy brain tissues with a few undergoing mitotic catastrophe (triple-positive cells) after irradiation. (**a**) White square indicates area covering both irradiated and non-irradiated tumor that is shown at higher magnification in (**d**). (**b**) Entire transverse section with immunofluorescence staining with white square indicating tumor area that was not irradiated (magnified in (**c**)). The transition between irradiated and non-irradiated tumor is marked with a dotted line. (**c**) Area between tumor and NHB that was irradiated; higher magnification image is shown in (**e**). Scale bar represents 40 μm. (**B**) Example of triple fluorescence staining of a tumor cell undergoing mitotic catastrophe. GL261 section (Figure 1A) was triple stained with anti-γ-H2AX/-CC3/-pHH3 antibodies and counter-stained with DAPI. Scale bar represents 10 μm. A few triple-positive tumor cells undergoing mitotic catastrophe were found, one of which is shown. The three fluorescence markers were sequentially added to the same field to better display the triple-positive cell.

To determine the effect of radiation on a mouse brain with an intracranial tumor, we irradiated only a portion of the tumor and adjacent brain tissue using conformal irradiation delivered from a small animal radiation research platform (SARRP) [5,6]. We expected that the tumor would serve as a positive control for the immunostaining procedure with a more robust DDR relative to the healthy brain. After one hour, the mouse was perfused, and the brain collected and sectioned, which was followed by multiplex immunostaining with antibodies against γ-H2AX (marking DNA double-strand breaks), cleaved caspase 3 (CC3; marking apoptosis), and phospho-(S10)-histone H3 (pHH3; marking mitotic cells). Together, these antibodies identify cells undergoing mitotic catastrophe (MC) after irradiation, which we have previously shown to occur in combination with ATMi’s in vitro [5] (Figure 1). We assumed that the irradiated tumor would show a more pronounced DDR and we would perhaps be able to identify cells undergoing MC [5]. Indeed, we were able to find a strong γ-H2AX response in the half of the tumor that was irradiated (red stained cells), indicating high levels of DNA double-strand breaks (Figure 1A). We also identified cells undergoing mitosis (pHH3+), dying cells (CC3+), as well as double-stained cells but very few triple-stained cells (4/51,447; <0.008%) in the irradiated tumor. However, there was only one triple-stained cell in the unirradiated tumor area (Figure 1B, Supplementary Table S1), suggesting that MC increases after radiation as expected but is a rare event. The portion of the field of the normal healthy brain (NHB) that was irradiated had cells with γ-H2AX foci and pHH3+ mitotic cells, and very few CC3+ ones. However, no triple-stained cells were detected at 1 h post-irradiation. Radiation increased the number of cells with γ-H2AX foci 100–150-fold in both tumor and NHB tissues as expected, whereas the CC3+ levels were unexpectedly reduced, by about 25–60% in both. One reason could be that radiation might already have killed many cells by the first hour post-irradiation. The opposite effect was seen when mitotic cells (pHH3+) were examined. While the levels went down 8-fold in the tumor after irradiation, perhaps as expected, due to triggering a G2/M checkpoint arrest, they went up several fold in the NHB. We refrain from speculating on the possible reason(s) for this result since we did not identify which subpopulation(s) were affected in the NHB. In vivo, healthy brain apoptosis occurs and peaks around 4 h after 20 Gy of radiation [17], a finding we previously documented with ATMi’s and 2 Gy of radiation [5]. The result from this experiment established the conditions for detecting DDR markers using multiplex immunofluorescence that sets the stage for studying radiation and ATMi effects on the mouse brain. Since this experiment did not cover a window spanning several hours and days after irradiation that could have generated more information, we decided to carry out a time-course experiment on irradiated mouse brains with and without an ATMi.

### 2.2. Time Course of Radiation-Induced Apoptosis in Healthy Brains

The mice were orally dosed with vehicle or AZD1390 [6], a clinical ATMi candidate that is currently in a human trial (NCT03423628), and then conformally irradiated (5 Gy) in a 5 × 5 mm field in the right hemisphere. The brains were collected at 4 h, 1 week, and 1 month after irradiation. Thus, each transverse brain section had an internal control, i.e., (1) unirradiated/irradiated and (2) unirradiated/irradiated in the presence of AZD1390. We then immunostained the brain sections with anti-γ-H2AX, -CC3, and -GFAP (marking gliosis) antibodies (Supplementary Figure S1), and semi-quantified the number of positive cells after IHC/DAB staining, which is summarized in Table 1. Briefly, and as expected, the number of γ-H2AX-positive cells increased after radiation and peaked at 4 h, subsided at 1 week, and disappeared by 1 month. With the AZD1390 treatment, the γ-H2AX levels were clearly reduced at 4 h, suggesting a partial inhibition due to ATM kinase activity, and were no longer detectable at 1 week, in line with that other PIKK-related kinases such as ATR and DNA-PK also phosphorylate H2AX at S139 [22]. On the other hand, the number of CC3-positive cells was moderate at 4 h and gone at 1 week and with the addition of AZD1390, the number of CC3-positive cells increased compared to 1 week after radiation alone. It is important to note that cells positive for CC3 staining will disappear, especially if apoptosis occurs during mitosis, which only lasts about an hour. Furthermore, in this experiment we did not distinguish between CNS sub-populations or whether cells were mitotic or terminally differentiated. Finally, when examining possible inflammatory responses (gliosis, activated microglial/astrocytes, and GFAP+ staining), we barely noticed any signs of inflammation with the radiation alone treatment except at 1 month, whereas dosing with AZD1390 and radiation seemed to elevate gliosis further (Supplementary Figure S1C).

### 2.3. ATM Kinase Inhibition Protects against Radiation-Induced Neuronal Apoptosis

**Figure 2.**
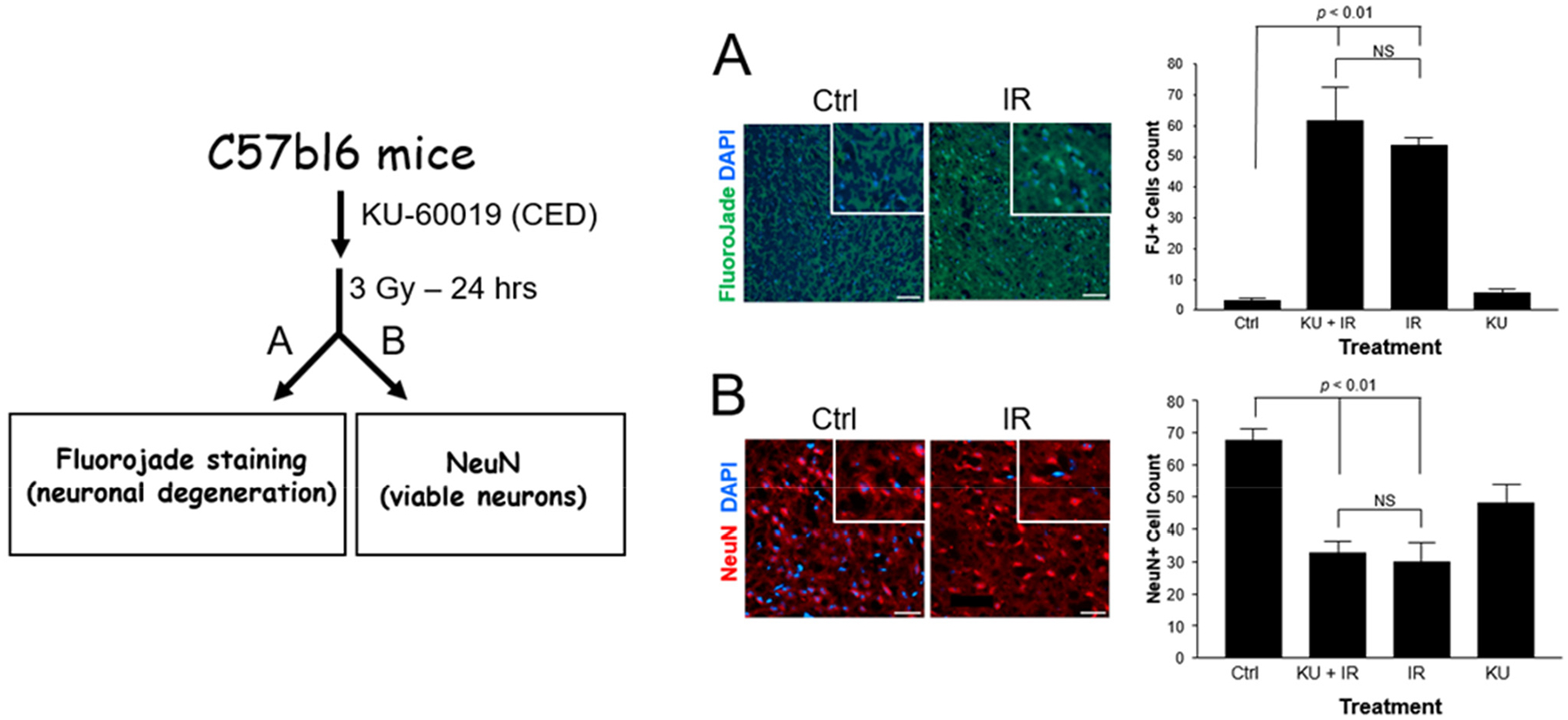
ATMi does not add to radiation-induced neuronal degeneration and cell death. Experimental outline (left). C57bl6 mice without tumors were injected with KU-60019 by CED followed by 137-Cs irradiation (3 Gy) and 24 h later, the brains were perfused and fixed. Coronal sections were obtained close to the CED injection site in the cerebral cortex of treated mice and processed as described in the Materials and Methods. (A) Staining with Fluorojade B and DAPI indicates degenerating neurons. (B) Immunostaining with anti-NeuN antibody and DAPI. Ctrl, unirradiated control; KU, KU-60019; IR, 3 Gy irradiation. Scale bar represents 100 μm. p-values are indicated. NS, non-significant.

To determine the effects of inhibiting ATM kinase on the mouse CNS, and more specifically on neurons, we administered an early ATM inhibitor (ATMi), KU-60019 [8,9], by convection-enhanced delivery (CED) [7], immediately followed by a single 3 Gy dose of conformal radiation to the brain using the SARRP. Twenty-four hours later, the mice were perfused and brains then processed for immunofluorescence imaging. To identify degenerating neurons, we used Fluorojade B (FJ) staining [23] and counter-stained with DAPI nuclear stain. Quantifying FJ+/DAPI cells in multiple sections and fields showed that neuronal degeneration after irradiation increased >20-fold over unirradiated brain tissue (*p* < 0.01). However, KU-60019 did not significantly add to radiation toxicity (Figure 2A). To corroborate this result, we quantified the number of neurons using immunostaining (NeuN+) of sections from control and irradiated mice (Figure 2B). Radiation reduced the number of neurons by ~60% (*p* < 0.01) at 24 h regardless of treatment with the ATMi. In this assay, the ATMi alone seemed to reduce the number of neurons by ~30%, a result which needs to be interrogated further since we have not seen such pronounced reduction when another ATMi (AZD1390) was administered by oral gavage. It is possible that the reduction in the number of neurons is the result of physical damage resulting from the process of inserting the cannula and the delivery by CED [7,24,25], an effect that might not be as evident when combined with radiation.

As a follow-up experiment to determine the effects of radiation and ATM inhibition on a mouse without a tumor, we re-examined the 4 h sections from the time-course experiment described above after staining with Akoya’s Opal Polaris 7 Color IHC Multiplex Detection kit; the slides were scanned and viewed in PhenoChart and the cell numbers were quantified using QuPath [26]. We analyzed the mouse brain sections in the irradiated and unirradiated areas treated with and without the ATM kinase inhibitor to determine the response in neurons (NeuN+) and changes in a combination of biomarkers: phosphorylated KRAB-associated protein 1 (pKAP1; marks the DDR, which is dependent on ATM), phosphorylated histone H2AX (γ-H2AX; marks double-stranded DNA breaks but is not a specific ATM kinase target), phosphorylated histone H3 (pHH3; marks cells in mitosis), and cleaved caspase 3 (CC3; marks cells undergoing apoptosis). Additionally, we looked for NeuN+ cells co-stained with γ-H2AX, pKAP1, and CC3, i.e., DDR+ neurons undergoing apoptosis [20] in response to radiation and AZD1390 (Supplementary Figure S2). We found that over a period of 4 h after 5 Gy of irradiation, AZD1390 protected mouse neurons from dying by 90% (Table 2, Supplementary Table S2). It can clearly be seen that AZD1390 blocked KAP1 phosphorylation after 5 Gy (NeuN:pKAP1 column) demonstrating AZD1390’s specificity for the ATM kinase. Apoptosis in neurons (NeuN:CC3 column) is known to occur spontaneously in the brain, events that should be subtracted from those seen after treatment to truly reflect the impact of AZD1390. Spontaneous apoptosis likely results from reactive-oxygen species, etc. In doing so, all NeuN columns align at approximately 90% inhibition of radiation-induced neuronal apoptosis by AZD1390 regardless of co-marker combination. Altogether, these results suggest that ATM kinase negatively regulates the survival of neurons after radiation.

## 3. Discussion

Our interim study using several ATMi’s and immunological assays showed that neurons are protected against radiation-induced apoptosis for up to several hours after radiation. Our result is in line with the conclusions from several previous rodent in vivo and in vitro studies demonstrating that when ATM is absent or inhibited, neurons are protected from radiation-induced apoptosis [17–20]. Other models, such as Drosophila, have been used to investigate the role of ATM in the CNS and whether neurons depend on ATM for cell death; these results agree with the results from rodents even though there are some differences [27–30]. Additionally, AZD1390 promotes significant neuron neurite outgrowth of dorsal root ganglion in mice [28]. Genetic and small molecule inhibition of ATM in mouse and Drosophila models of Huntington’s disease protect striatal neurons against hydrogen peroxide-generated stress and the triggering of a neurotoxic DDR [31], again supporting the idea that ATM negatively regulates the survival of neurons when challenged by radiation and DNA damage. Importantly, every kinase, including ATM, has a designated phosphatase that, at some point, dephosphorylates the target(s) when cellular conditions change. Protein phosphatase 2A (PP2A) is considered a major co-regulator of the DDR by dephosphorylating many proteins phosphorylated by ATM kinase thereby reversing the DDR back to normalcy [32–34]. PP2A also plays a critical role in neurodegenerative diseases such as Alzheimer’s where it is downregulated or inhibited, resulting in increased Tau phosphorylation [35]. Therefore, PP2 and ATM need to be considered together when it comes to DDR effects on the brain. A remaining question is whether neurons recover from irradiation and survive after the ATMi is no longer present. In other words, how long would ATM kinase need to be inhibited after radiation for neurons to survive? Therefore, it is important to carefully examine the long-term effects of radiation with and without AZD1390 over several months to determine the impact on neurodegeneration.

## 4. Material and Methods

### 4.1. Animals

C57bl6 mice were obtained from the Jackson Laboratory and bred in the Virginia Commonwealth University Massey Comprehensive Cancer Center (MCCC) vivarium. The experiments were conducted following a protocol (AM10197) approved by the VCU Institutional Animal Care and Use Committee (IACUC). The animals were maintained in an environment with constant temperature and humidity on a 12 h light/12 h dark schedule, and with ad libitum access to water and chow.

### 4.2. Cells, Cell Culture, and Treatments

Mouse GL261 glioma cells were cultured as monolayer in DMEM supplemented with 10% fetal bovine serum and penicillin/streptomycin. Intra-cranial cell injections were performed as described previously [5–7]. Mice were injected with ~10^5^ GL261 glioma cells to generate intra-cranial tumors at the age of 8–10 weeks and irradiated approximately 3 weeks later. The irradiation of the mice was conducted using an MDS Nordion Gammacell 40 research irradiator with a Cs-137 source, delivering a dose rate of 1.05 Gy/min. The mice were shielded with lead blocks so that only the brain was irradiated. Mice with or without GL261 gliomas were conformally irradiated using a small animal radiation research platform (SARRP), as described previously [5–7]. The SARRP delivers up to 225 kV with a copper filter (0.15 mm) at an approximate dose rate of 1 Gy/min.

### 4.3. Antibodies and Reagents

The antibodies used for immunohistochemistry were rabbit anti-γ-H2AX (Millipore), NeuN, cleaved caspase-3, Alexa Fluor 594 goat anti-mouse immunoglobulin G (IgG), Alexa Fluor 594 goat anti-rabbit immunoglobulin G (IgG), Alexa Fluor 488, DAPI stain, and Fluorojade B (Millipore). Matrigel (BD Biosciences, Franklin Lakes, NJ, USA) was used for tumor cell implantation. KU-60019 and AZD1390 were purchased from Selleck Chemicals LLC (Houston, TX, USA).

### 4.4. Imaging and Microscopy

Following KU-60019 administration by convection-enhanced delivery(CED) and whole brain irradiation from a Nordion Gammacell 40 137-Cs source, the mice were transcardially perfused with 3% paraformaldehyde after 3 h, followed by post-fixing, as previously described [7]. The brains were cut coronally through the CED injection site (2 mm left lateral and 1 mm posterior to the bregma to a depth of 3 mm), and the posterior portion was embedded in Tissue-Tek OCT compound (Sakura Finetek, Torrence, CA, USA), frozen on dry ice, and stored at −20 ^°^C. Coronal cryo-sectioning was carried out on a Leica CM1850 cryostat to a 6 μm thickness and the slides were stored frozen until staining. The sections were permeabilized with 1% Triton-X-100/PBS before exposure to rabbit anti-NeuN antibody (Cell Signaling Technology, Inc., Danvers, MA, USA) followed by secondary Alexa Fluor-594 goat-anti rabbit antibody and DAPI. Fluorojade B (FJ) staining was carried out on the frozen sections. The sections were treated sequentially with 1% NaOH in 80% ethanol, potassium permanganate to suppress background fluorescence, stained with FJ, washed with distilled water, cleared with Histoclear, and mounted with coverslips using Permount (Fisher Scientific, Waltham, MA, USA). For co-staining with antibodies and FJ, the sections were exposed to the antibodies first, followed by incubation in potassium permanganate for 5 min and then treatment with FJ for 5 min. The sections were then rinsed in distilled water, stained with DAPI, and mounted with coverslips. The sections were imaged using the Ariol SL-50 system (Applied Imaging Corp, San Jose, CA, USA).

For the IHC and multiplex immunofluorescence experiments, the mice were irradiated with 5 Gy (either 5 × 5 mm or 3 ×9 mm collimator) to the frontal right hemisphere using the SARRP and the left hemisphere was designated as the untreated control for radiation and AZD1390, respectively. AZD1390 was administered p.o. (20 mg/kg) 1 h prior to the irradiation procedure [6]. The brains were collected at 4 h, 1 week, and 1 month after treatment following perfusion with 4% formaldehyde, embedding in paraffin, transverse sectioning, treatment with EDTA for antigen retrieval, and then incubated with hydrogen peroxide for 5 min to quench any endogenous peroxidase activity. HRP-IHC was performed using Bond RX (Leica Biosystems, Buffalo Grove, IL, USA). Rabbit anti-γ-H2AX (H2A.X S139 (20E3) #9718) (diluted 1:250) and cleaved caspase 3 (#9664) (diluted 1:500) antibodies, both from Cell Signaling, and mouse anti-GFAP antibody (Abcam, Waltham, MA, USA; #4648) (diluted 1:250) were applied followed by incubation with goat anti-rabbit IgG-HRP and 3,3^*′*^-diaminobenzidine tetrahydrochloride hydrate (DAB). The sections were counter-stained with hematoxylin. For the multiplex Akoya immunofluorescence staining, the following rabbit antibodies (Cell Signaling) and their dilutions and respective OPAL stains were used: anti-phospho-(S10) histone H3 (1:250)/Opal 480; proliferating cell nuclear antigen (1:2000)/Opal 520; phospho-(S824) KRAB-associated protein-1 (1:250)/Opal 570; cleaved caspase 3 (1:250)/Opal 620; phospho-(S139) γ-H2AX (1:250)/Opal 690; NeuN (1:250)/Opal 780; and DAPI nuclear stain (1 μg/mL).

## 5. Statistics

Unpaired, two-tailed t test or one-way ANOVA was performed on the data sets (with three or more replicates) using GraphPad Prism 3.0 (Graphpad Software, Inc., Boston, MA, USA).

## Supplementary Material

1

## Figures and Tables

**Table 1.** Immunostaining of partially irradiated mouse brains. C57bl6 mice were partially irradiated (5 Gy) using SARRP with a 5 × 5 mm collimator and the brains collected at 4 h, 1 week, and 1 month after irradiation. Sections were stained with anti-γ-H2AX, -CC3, and -GFAP antibodies followed by horseradish peroxidase (HRP/DAB) IHC and counter-staining with hematoxylin. The cells were assessed for DAB+ and semi-quantitatively scored as absent (−), few (+), moderate (++), or dense (+++).

Treatment		Antibody											
		γ-H2AX				CC3				GFAP (gliosis)			
5 Gy		-		+		-		+		-		+	
AZD1390		-	+	-	+	-	+	-	+	-	+	-	+
Time	4 hrs	-	-	+++	+(+)	-	-	+(+)	++	-	-	-	-
	1 week	-	-	++	-	-	-	-	−/+	-	-	-	-
	1 month	-	-	-	-	-	-	-	-	-	-	−/+	++

**Table 2.** Immunostaining of a partially irradiated mouse brain. C57bl6 mice were partially irradiated (5 Gy) in the right hemisphere using SARRP and a 5 × 5 mm collimator and the brains were collected at 4 h after radiation. The sections were stained with anti-NeuN, γ-H2AX, pKAP1, and CC3 antibodies following the Akoya multiplex immunostaining protocol. Cell events were quantified using the QuPath image analysis software (QuPath (0.1.2)).

		DDR in NeuN+ cells			DDR-Induced Apoptosis		
Treatment	Cell Numbers	Antibody					
		NeuN: pKAP1	NeuN: Y-H2AX: pKAP1	NeuN: CC3	NeuN: CC3: pKAP1	NeuN: CC3: Y-H2AX	NeuN: CC3: Y-H2AX: pKAP1
Untreated	8480	0	0	447	0	10	0
AZD1390	9064	1	0	104	0	0	0
5 Gy	9504	89	30	918	184 (1.9%)	108 (1.1%)	161 (1.7%)
AZD1390 + 5 Gy	9425	1	5	458	0	14 (0.15%)	16 (0.17%)

## Data Availability

The original contributions presented in the study are included in the article/supplementary material, further inquiries can be directed to the corresponding author.

## References

[1] Shiloh Y; Ziv Y The ATM protein kinase: Regulating the cellular response to genotoxic stress, and more. Nat. Rev. Mol. Cell Biol 2013, 14, 197–210. 23847781

[2] Lavin MF Ataxia-telangiectasia: From a rare disorder to a paradigm for cell signalling and cancer. Nat. Rev. Mol. Cell Biol 2008, 9, 759–769. 18813293 10.1038/nrm2514

[3] Herrup K ATM and the epigenetics of the neuronal genome. Mech. Ageing Dev 2013, 134, 434–439. 23707635 10.1016/j.mad.2013.05.005 PMC3791148

[4] Rodier F; Coppe JP; Patil CK; Hoeijmakers WA; Munoz DP; Raza SR; Freund A; Campeau E; Davalos AR; Campisi J Persistent DNA damage signalling triggers senescence-associated inflammatory cytokine secretion. Nat. Cell Biol 2009, 11, 973–979. 19597488 10.1038/ncb1909 PMC2743561

[5] Karlin J; Allen J; Ahmad SF; Hughes G; Sheridan V; Odedra R; Farrington P; Cadogan EB; Riches LC; Garcia-Trinidad A; Orally Bioavailable and Blood-Brain Barrier-Penetrating ATM Inhibitor (AZ32) Radiosensitizes Intracranial Gliomas in Mice. Mol. Cancer Ther 2018, 17, 1637–1647. 29769307 10.1158/1535-7163.MCT-17-0975 PMC6072596

[6] Durant ST; Zheng L; Wang Y; Chen K; Zhang L; Zhang T; Yang Z; Riches L; Trinidad AG; Fok JHL; The brain-penetrant clinical ATM inhibitor AZD1390 radiosensitizes and improves survival of preclinical brain tumor models. Sci. Adv 2018, 4, eaat1719. 29938225 10.1126/sciadv.aat1719 PMC6010333

[7] Biddlestone-Thorpe L; Sajjad M; Rosenberg E; Beckta JM; Valerie NC; Tokarz M; Adams BR; Wagner AF; Khalil A; Gilfor D; ATM kinase inhibition preferentially sensitizes p53-mutant glioma to ionizing radiation. Clin. Cancer Res. Off. J. Am. Assoc. Cancer Res 2013, 19, 3189–3200. 10.1158/1078-0432.CCR-12-3408 PMC3687028 23620409

[8] Golding SE; Rosenberg E; Adams BR; Wignarajah S; Beckta JM; O’Connor MJ; Valerie K Dynamic inhibition of ATM kinase provides a strategy for glioblastoma multiforme radiosensitization and growth control. Cell Cycle 2012, 11, 1167–1173. 22370485 10.4161/cc.11.6.19576 PMC3335919

[9] Golding SE; Rosenberg E; Valerie N; Hussaini I; Frigerio M; Cockcroft XF; Chong WY; Hummersone M; Rigoreau L; Menear KA; Improved ATM kinase inhibitor KU-60019 radiosensitizes glioma cells, compromises insulin, AKT and ERK prosurvival signaling, and inhibits migration and invasion. Mol. Cancer Ther 2009, 8, 2894–2902. 19808981 10.1158/1535-7163.MCT-09-0519 PMC2761990

[10] Golding SE; Morgan RN; Adams BR; Hawkins AJ; Povirk LF; Valerie K Pro-survival AKT and ERK signaling from EGFR and mutant EGFRvIII enhances DNA double-strand break repair in human glioma cells. Cancer Biol. Ther 2009, 8, 730–738. 19252415 10.4161/cbt.8.8.7927 PMC2863288

[11] Golding SE; Rosenberg E; Neill S; Dent P; Povirk LF; Valerie K Extracellular signal-related kinase positively regulates ataxia telangiectasia mutated, homologous recombination repair, and the DNA damage response. Cancer Res. 2007, 67, 1046–1053. 17283137 10.1158/0008-5472.CAN-06-2371

[12] Golding SE; Rosenberg E; Khalil A; McEwen A; Holmes M; Neill S; Povirk LF; Valerie K Double strand break repair by homologous recombination is regulated by cell cycle-independent signaling via ATM in human glioma cells. J. Biol. Chem 2004, 279, 15402–15410. 14744854 10.1074/jbc.M314191200

[13] Chen J; Laverty DJ; Talele S; Bale A; Carlson BL; Porath KA; Bakken KK; Burgenske DM; Decker PA; Vaubel RA; Aberrant ATM signaling and homology-directed DNA repair as a vulnerability of p53-mutant GBM to AZD1390-mediated radiosensitization. Sci. Transl. Med 2024, 16, eadj5962. 38354228 10.1126/scitranslmed.adj5962 PMC11064970

[14] Stupp R; Mason WP; van den Bent MJ; Weller M; Fisher B; Taphoorn MJ; Belanger K; Brandes AA; Marosi C; Bogdahn U; Radiotherapy plus concomitant and adjuvant temozolomide for glioblastoma. N. Engl. J. Med 2005, 352, 987–996. 15758009 10.1056/NEJMoa043330

[15] Monje ML; Mizumatsu S; Fike JR; Palmer TD Irradiation induces neural precursor-cell dysfunction. Nat. Med 2002, 8, 955–962. 12161748 10.1038/nm749

[16] Monje ML; Palmer T Radiation injury and neurogenesis. Curr. Opin. Neurol 2003, 16, 129–134. 12644738 10.1097/01.wco.0000063772.81810.b7

[17] Herzog KH; Chong MJ; Kapsetaki M; Morgan JI; McKinnon PJ Requirement for Atm in ionizing radiation-induced cell death in the developing central nervous system. Science 1998, 280, 1089–1091. 9582124 10.1126/science.280.5366.1089

[18] Gosink EC; Chong MJ; McKinnon PJ Ataxia telangiectasia mutated deficiency affects astrocyte growth but not radiosensitivity. Cancer Res. 1999, 59, 5294–5298. 10537312

[19] Chong MJ; Murray MR; Gosink EC; Russell HR; Srinivasan A; Kapsetaki M; Korsmeyer SJ; McKinnon PJ Atm and Bax cooperate in ionizing radiation-induced apoptosis in the central nervous system. Proc .Natl. Acad. Sci. USA 2000, 97, 889–894. 10639175 10.1073/pnas.97.2.889 PMC15426

[20] Kruman II; Wersto RP; Cardozo-Pelaez F; Smilenov L; Chan SL; Chrest FJ; Emokpae R Jr.; Gorospe M; Mattson MP Cell cycle activation linked to neuronal cell death initiated by DNA damage. Neuron 2004, 41, 549–561. 14980204 10.1016/s0896-6273(04)00017-0

[21] Katyal S; McKinnon PJ DNA strand breaks, neurodegeneration and aging in the brain. Mech. Ageing Dev 2008, 129, 483–491. 18455751 10.1016/j.mad.2008.03.008 PMC3831510

[22] Burma S; Chen BP; Murphy M; Kurimasa A; Chen DJ ATM Phosphorylates Histone H2AX in Response to DNA Double-strand Breaks. J. Biol. Chem. 2001, 276, 42462–42467. 11571274 10.1074/jbc.C100466200

[23] Schmued LC Development and application of novel histochemical tracers for localizing brain connectivity and pathology. Brain Res. 2016, 1645, 31–35. 27155454 10.1016/j.brainres.2016.03.053

[24] Biddlestone-Thorpe L; Marchi N; Guo K; Ghosh C; Janigro D; Valerie K; Yang H Nanomaterial-mediated CNS delivery of diagnostic and therapeutic agents. Adv. Drug Deliv. Rev 2012, 64, 605–613. 22178615 10.1016/j.addr.2011.11.014 PMC3321130

[25] Khalsa JK; Cheng N; Keegan J; Chaudry A; Driver J; Bi WL; Lederer J; Shah K Immune phenotyping of diverse syngeneic murine brain tumors identifies immunologically distinct types. Nat. Commun. 2020, 11, 3912. 32764562 10.1038/s41467-020-17704-5 PMC7411074

[26] Bankhead P; Loughrey MB; Fernandez JA; Dombrowski Y; McArt DG; Dunne PD; McQuaid S; Gray RT; Murray LJ; Coleman HG; QuPath: Open source software for digital pathology image analysis. Sci. Rep 2017, 7, 16878. 29203879 10.1038/s41598-017-17204-5 PMC5715110

[27] Taylor MJ; Thompson AM; Alhajlah S; Tuxworth RI; Ahmed Z Inhibition of Chk2 promotes neuroprotection, axon regeneration, and functional recovery after CNS injury. Sci. Adv 2022, 8, eabq2611. 36103534 10.1126/sciadv.abq2611 PMC9473583

[28] Ahmed Z; Tuxworth RI The brain-penetrant ATM inhibitor, AZD1390, promotes axon regeneration and functional recovery in preclinical models of spinal cord injury. Clin. Transl. Med 2022, 12, e962. 35818848 10.1002/ctm2.962 PMC9274214

[29] Tuxworth RI; Taylor MJ; Martin Anduaga A; Hussien-Ali A; Chatzimatthaiou S; Longland J; Thompson AM; Almutiri S; Alifragis P; Kyriacou CP; Attenuating the DNA damage response to double-strand breaks restores function in models of CNS neurodegeneration. Brain Commun. 2019, 1, fcz005. 32954257 10.1093/braincomms/fcz005 PMC7425387

[30] Rimkus SA; Katzenberger RJ; Trinh AT; Dodson GE; Tibbetts RS; Wassarman DA Mutations in String/CDC25 inhibit cell cycle re-entry and neurodegeneration in a Drosophila model of Ataxia telangiectasia. Genes Dev. 2008, 22, 1205–1220. 18408079 10.1101/gad.1639608 PMC2335316

[31] Lu XH; Mattis VB; Wang N; Al-Ramahi I; van den Berg N; Fratantoni SA; Waldvogel H; Greiner E; Osmand A; Elzein K; Targeting ATM ameliorates mutant Huntingtin toxicity in cell and animal models of Huntington’s disease. Sci. Transl. Med 2014, 6, 268ra178. 10.1126/scitranslmed.3010523 25540325

[32] Chowdhury D; Keogh MC; Ishii H; Peterson CL; Buratowski S; Lieberman J gamma-H2AX dephosphorylation by protein phosphatase 2A facilitates DNA double-strand break repair. Mol. Cell 2005, 20, 801–809. 16310392 10.1016/j.molcel.2005.10.003

[33] Sule A; Golding SE; Ahmad SF; Watson J; Ahmed MH; Kellogg GE; Bernas T; Koebley S; Reed JC; Povirk LF; ATM phosphorylates PP2A subunit A resulting in nuclear export and spatiotemporal regulation of the DNA damage response. Cell. Mol. Life Sci 2022, 79, 603. 36434396 10.1007/s00018-022-04550-5 PMC9700600

[34] Li J; Chen J; Ricupero CL; Hart RP; Schwartz MS; Kusnecov A; Herrup K Nuclear accumulation of HDAC4 in ATM deficiency promotes neurodegeneration in ataxia telangiectasia. Nat. Med 2012, 18, 783–790. 22466704 10.1038/nm.2709 PMC3378917

[35] Sontag JM; Sontag E Protein phosphatase 2A dysfunction in Alzheimer’s disease. Front. Mol. Neurosci 2014, 7, 16. 24653673 10.3389/fnmol.2014.00016 PMC3949405

