# Supplementary material for: ATM Kinase Small Molecule Inhibitors Prevent Radiation-Induced Apoptosis of Mouse Neurons In Vivo
Source: Kinases Phosphatases. Author manuscript; Available in PMC 2025 Apr 9. (PMC11981642; doi:10.3390/kinasesphosphatases2030017)
Supplement: 1 [file NIHMS2067442-supplement-1.pdf]

Supplementary Materials

ATM kinase small molecule inhibitors prevent radiation-induced apoptosis of mouse neurons in vivo

Yüksel Aydar[1, †], Sanara S Rambukkanage[1, †], Lauren Brown[1, †], Juan Wang[1, †], Ji Sung Seo[1], Keming Li[1], Yong Cheng[1], Laura Biddlestone-Thorpe[1], Caila Boyd[1], Amrita Sule[1], and Kristoffer Valerie[1,2*]

[1]Department of Radiation Oncology, Massey Comprehensive Cancer Center[2], Virginia Commonwealth University, Richmond, Virginia, USA

Figure and Table legends

Figure S1. Immunohistochemistry of irradiated mouse brains after dosing with AZD1390 or not. Brains were collected at 4 hrs, 1 week, and 1 month after irradiation. Transverse brain sections were prepared with internal treatment controls, ie, unirradiated/irradiated and unirradiated/irradiated in the presence of AZD1390. Sections were stained with antibodies against **(A)** γ-H2AX, **(B)** CC3, and **(C)** GFAP (marking gliosis) as well as **(D)** H&E.

Figure S2. Brain sections of partially irradiated mice brains that were stained with antibodies and processed for Akoya multiplex immunofluorescence. The samples were extracted 4 hours after 5 Gy conformal radiation using SARRP. Mice administered vehicle (*top section*) or AZD1390 (*bottom section*) 1 hour prior to SARRP. Area A depicts unirradiated, B depicts irradiated, C depicts AZD1390, and D depicts irradiated after dosing with AZD1390. Antibodies pHH3 (turquoise), PCNA (green); staining control, pKAP-1 (yellow), CC3 (orange), γ-H2AX (red), NeuN (white), and DAPI (blue).

Table S1. Summary of multiplex immunostaining with antibodies against γ-H2AX, cleaved caspase 3, and phospho-(S10)-histone H3 , together identifying cells undergoing mitotic catastrophe in tumor and normal, healthy brain (NHB) after radiation.

Supplementary Fig. S1A

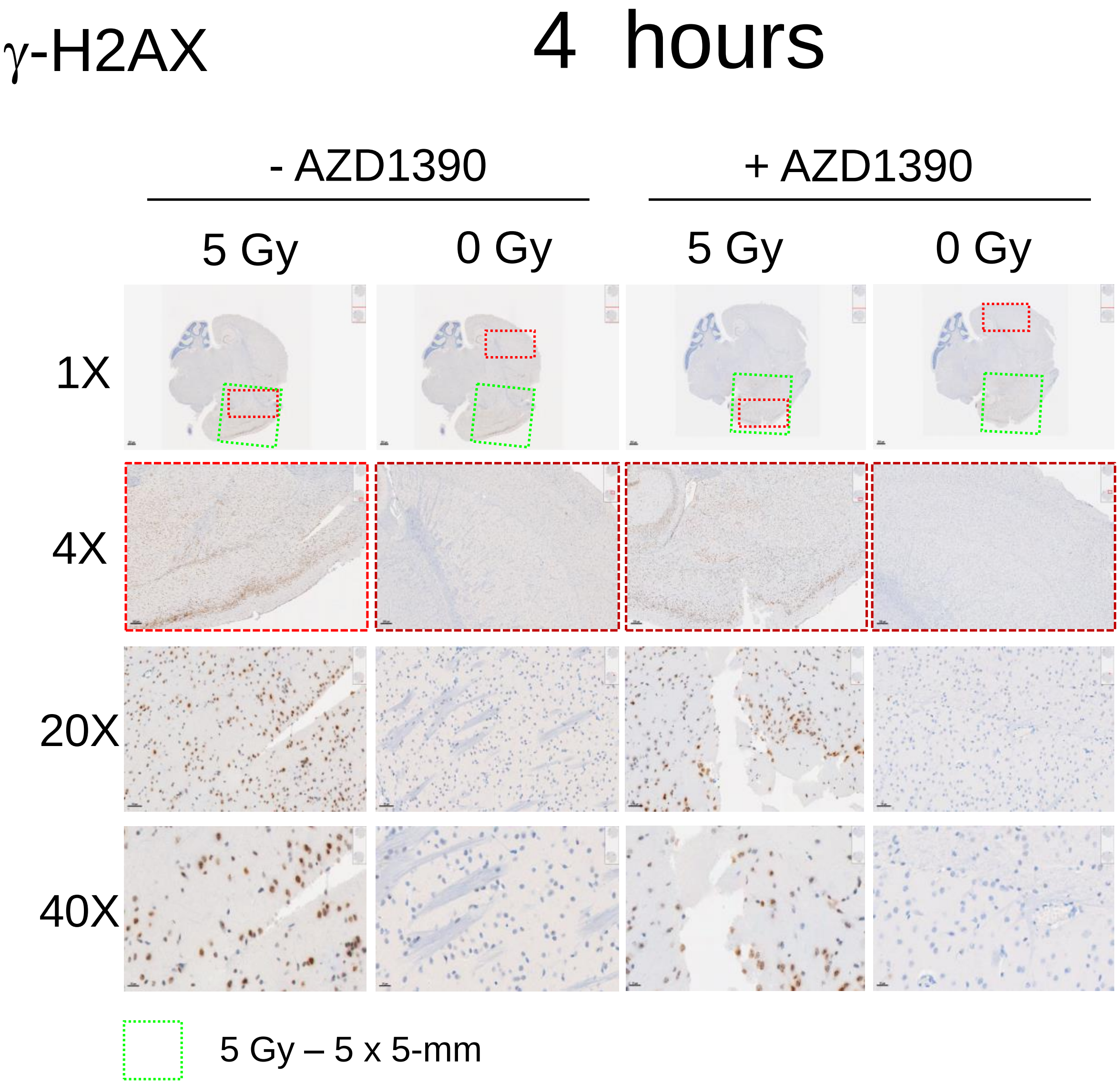

Supplementary Fig. S1A

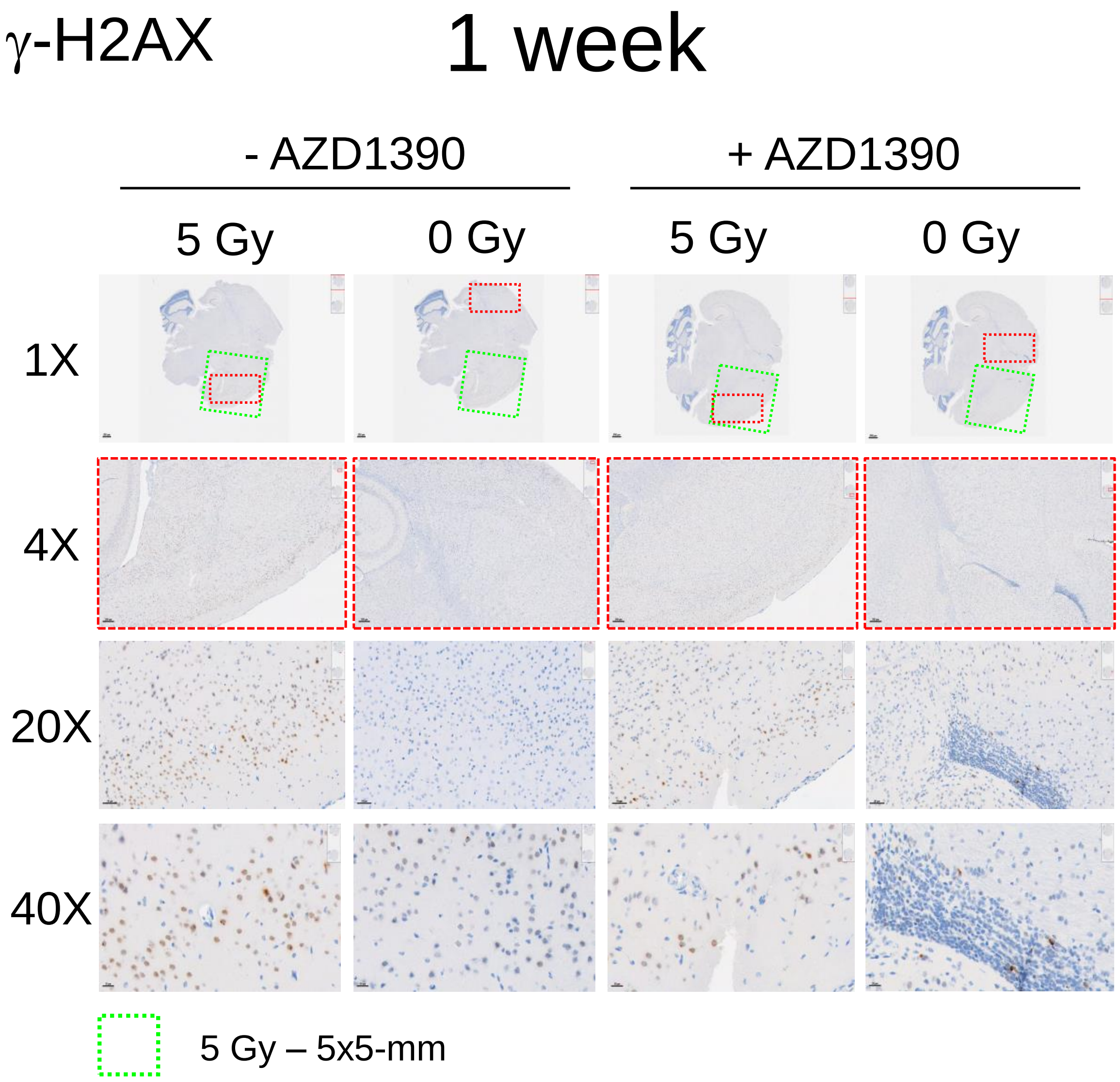

Supplementary Fig. S1A

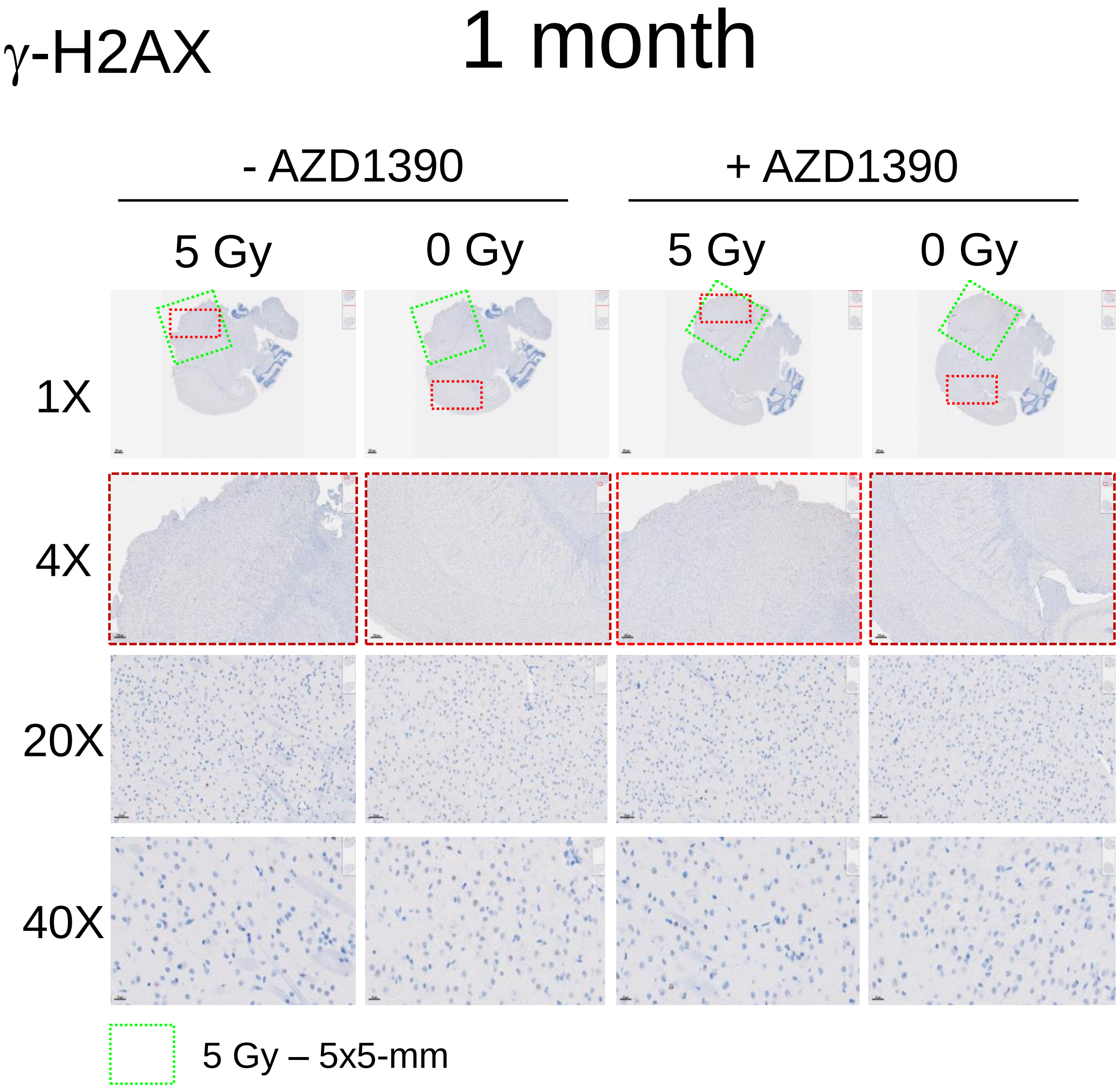

Supplementary Fig. S1B

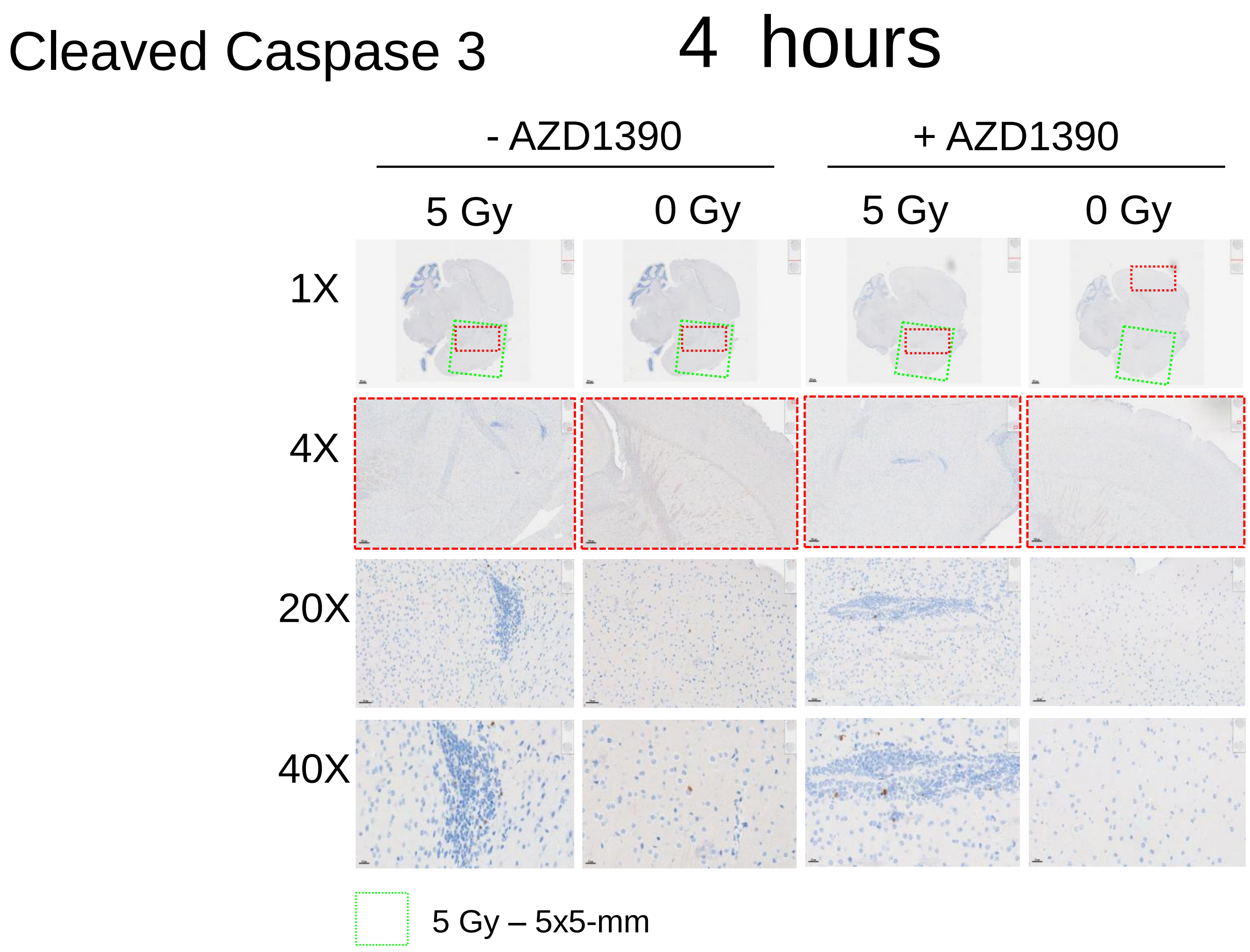

Supplementary Fig. S1B

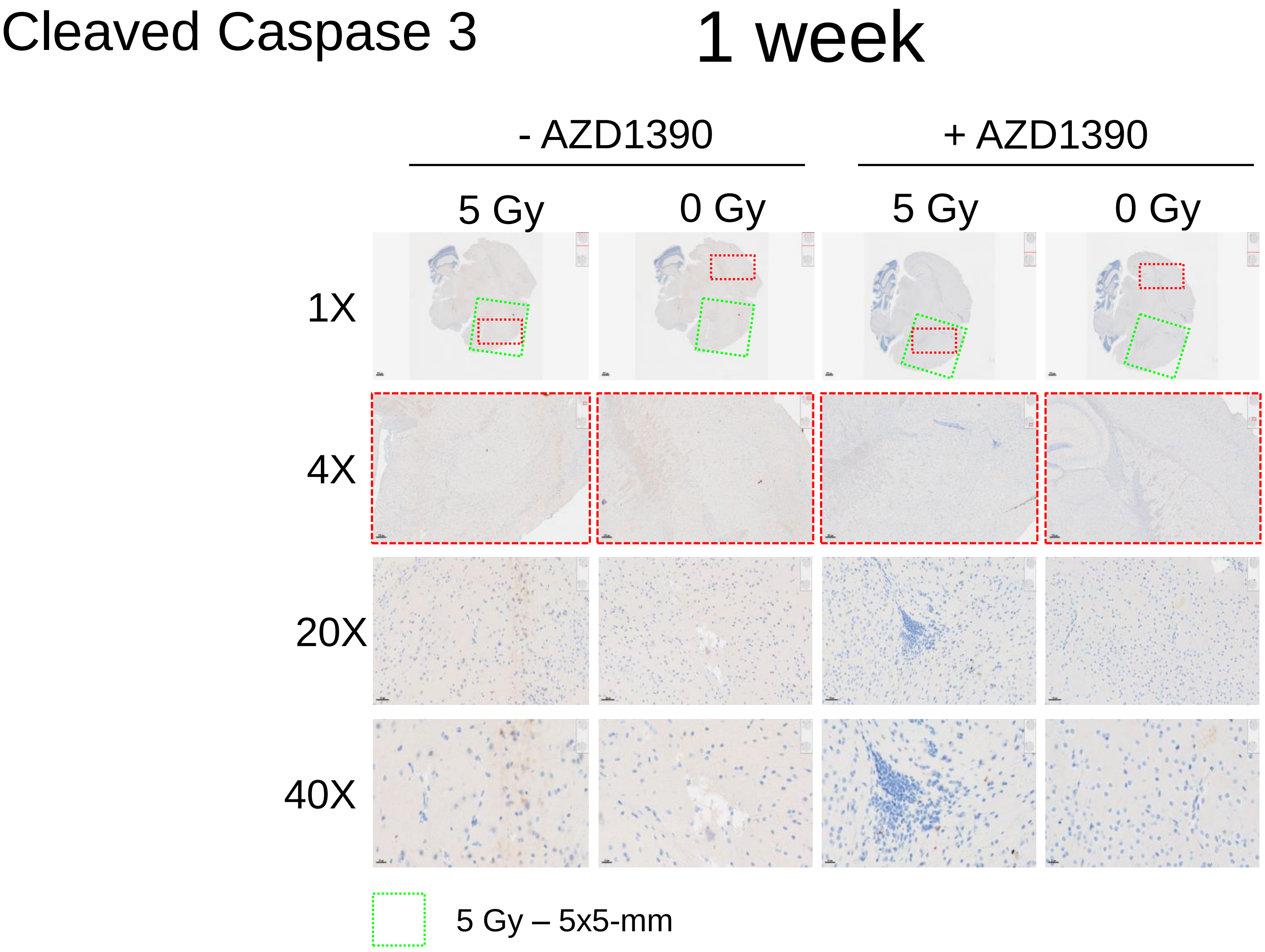

Supplementary Fig. S1B

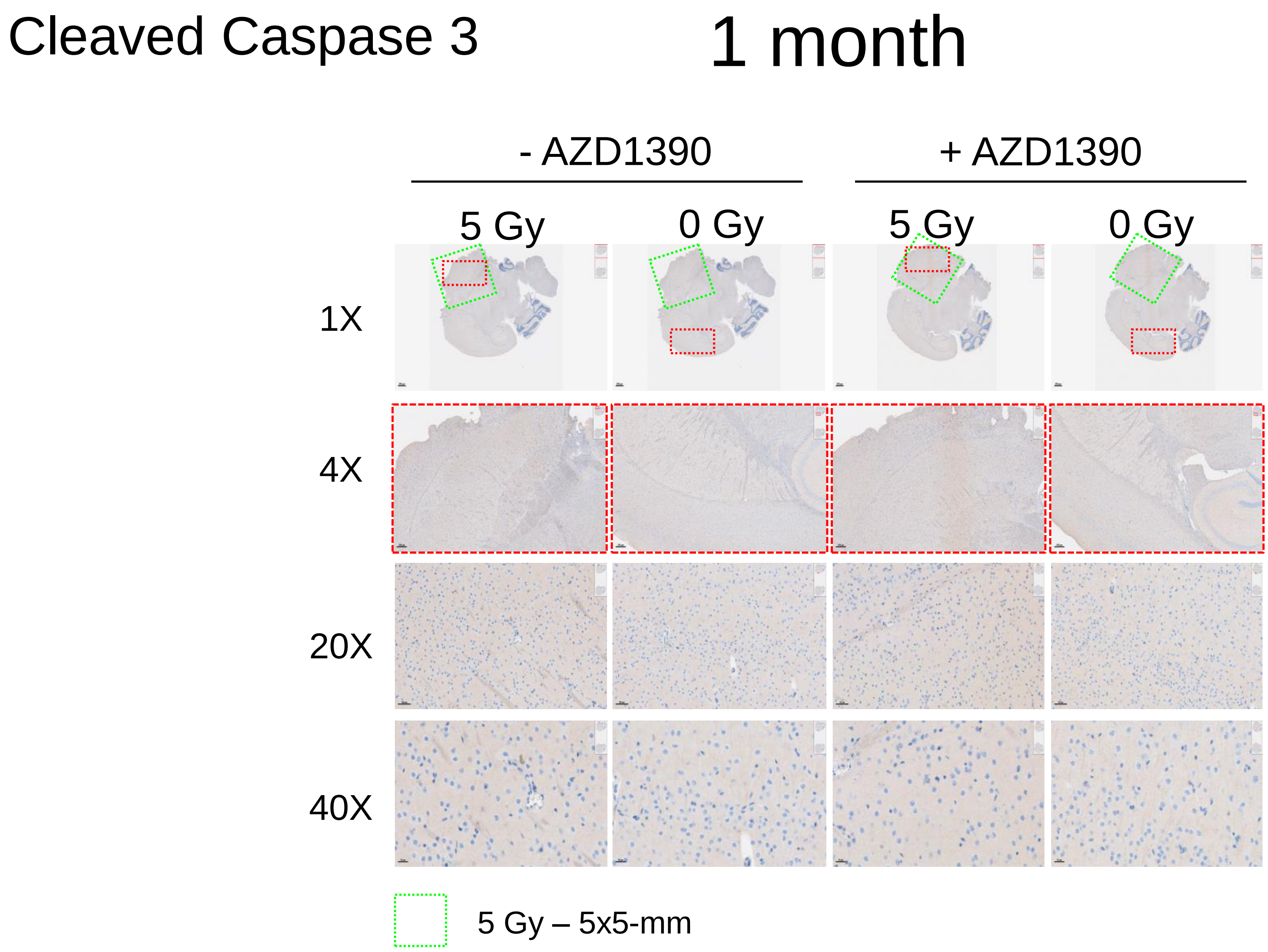

Supplementary Fig. S1C

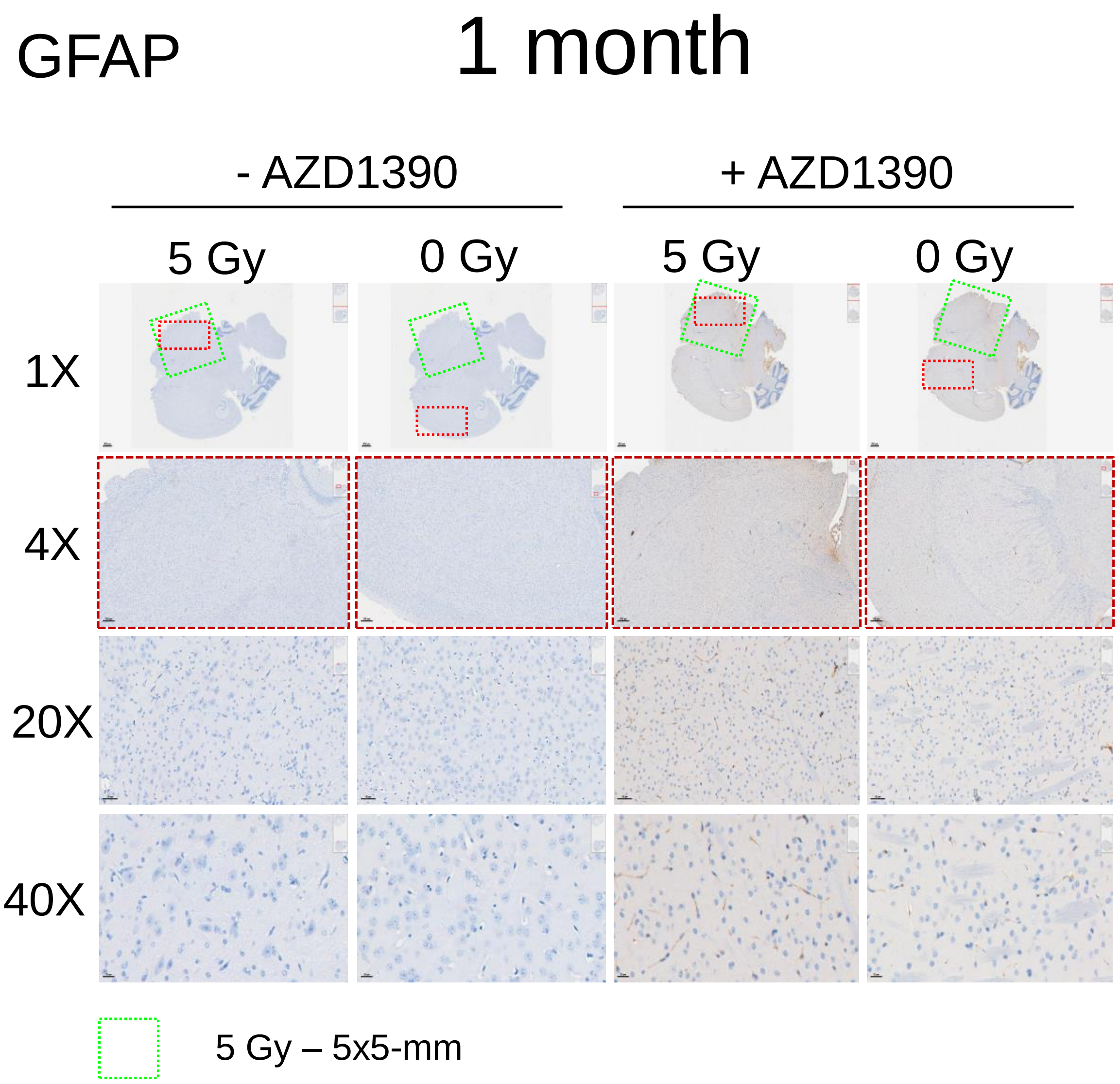

Supplementary Fig. S1D

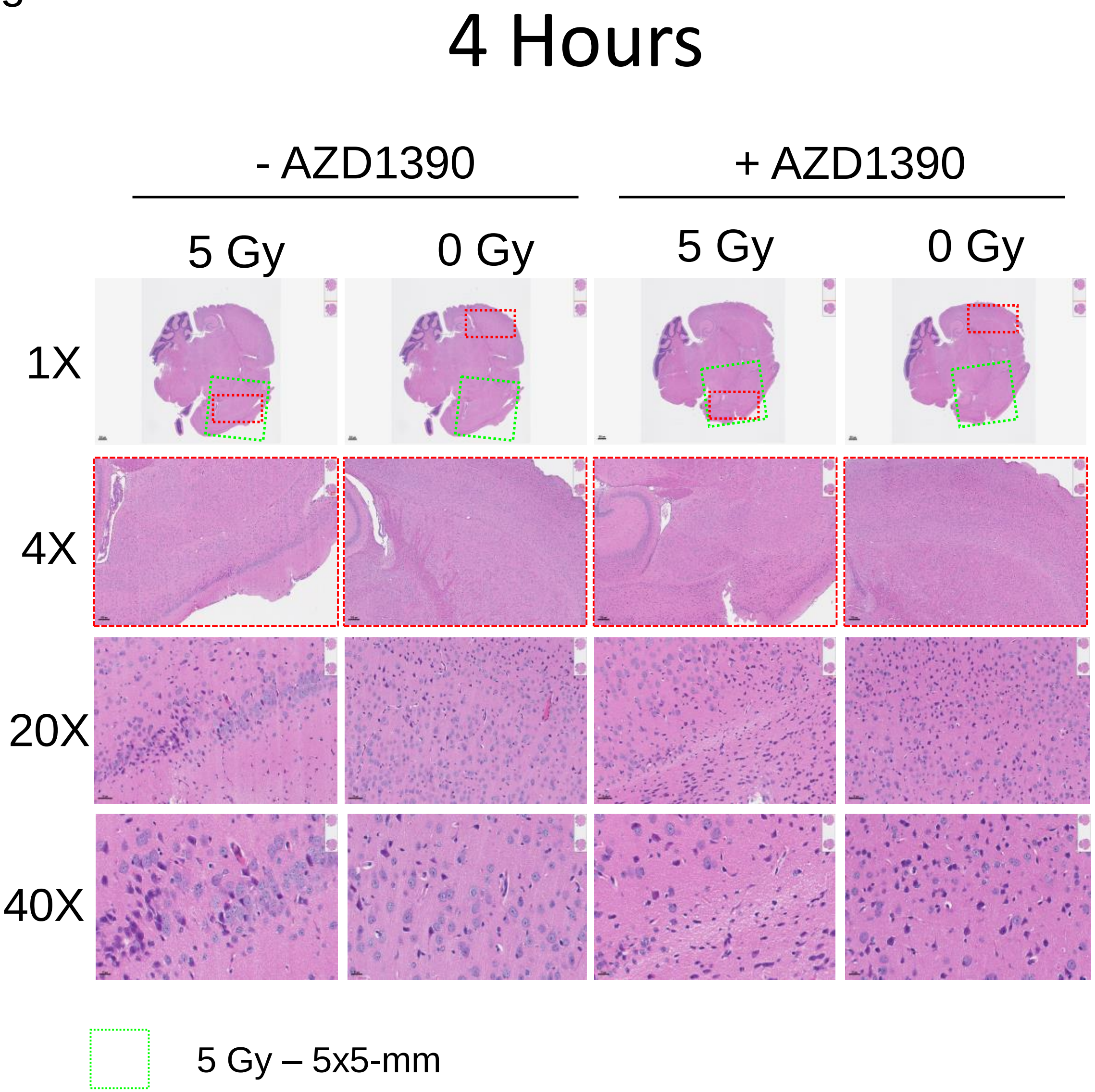

Supplementary Fig. S1D

1 Week

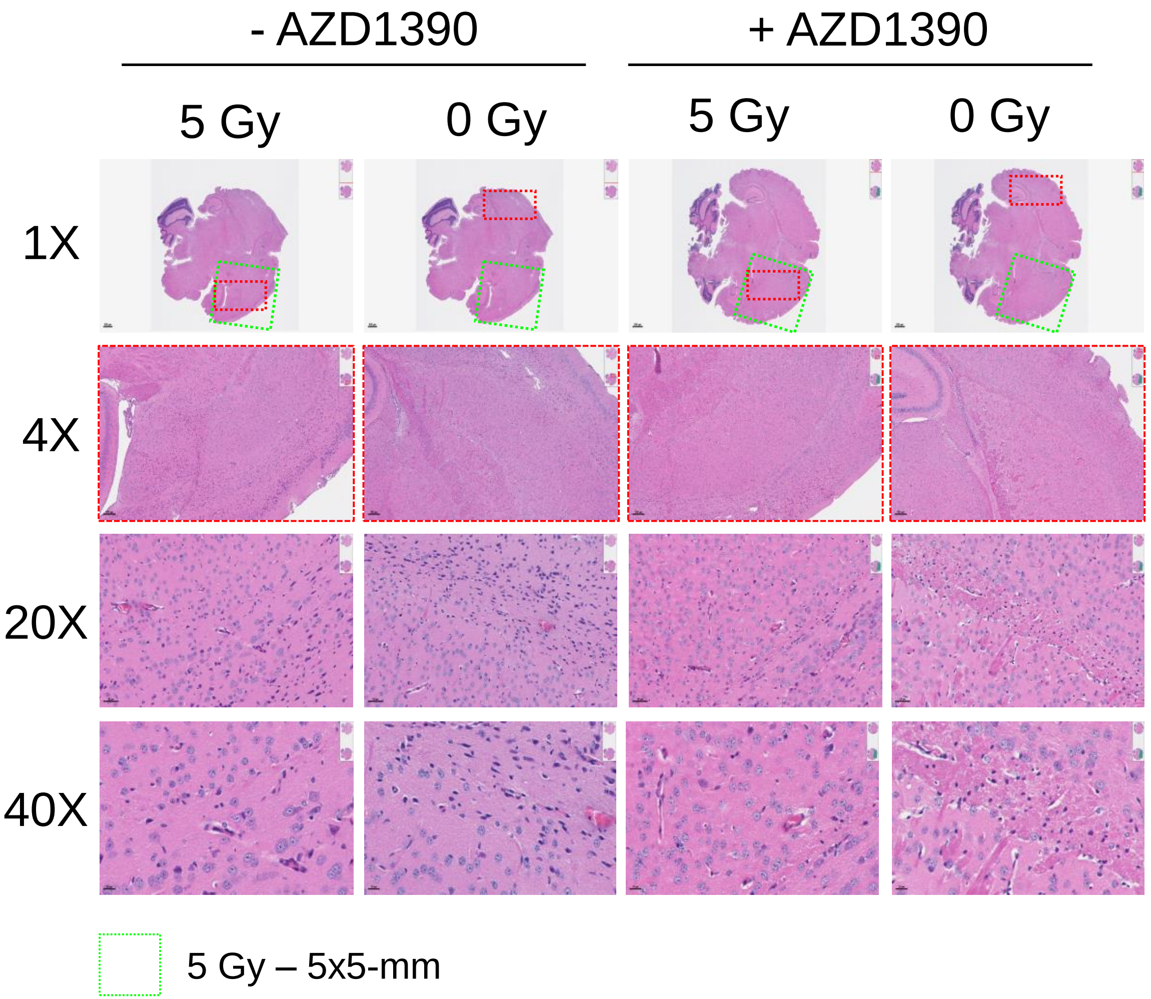

Supplementary Fig. S1D

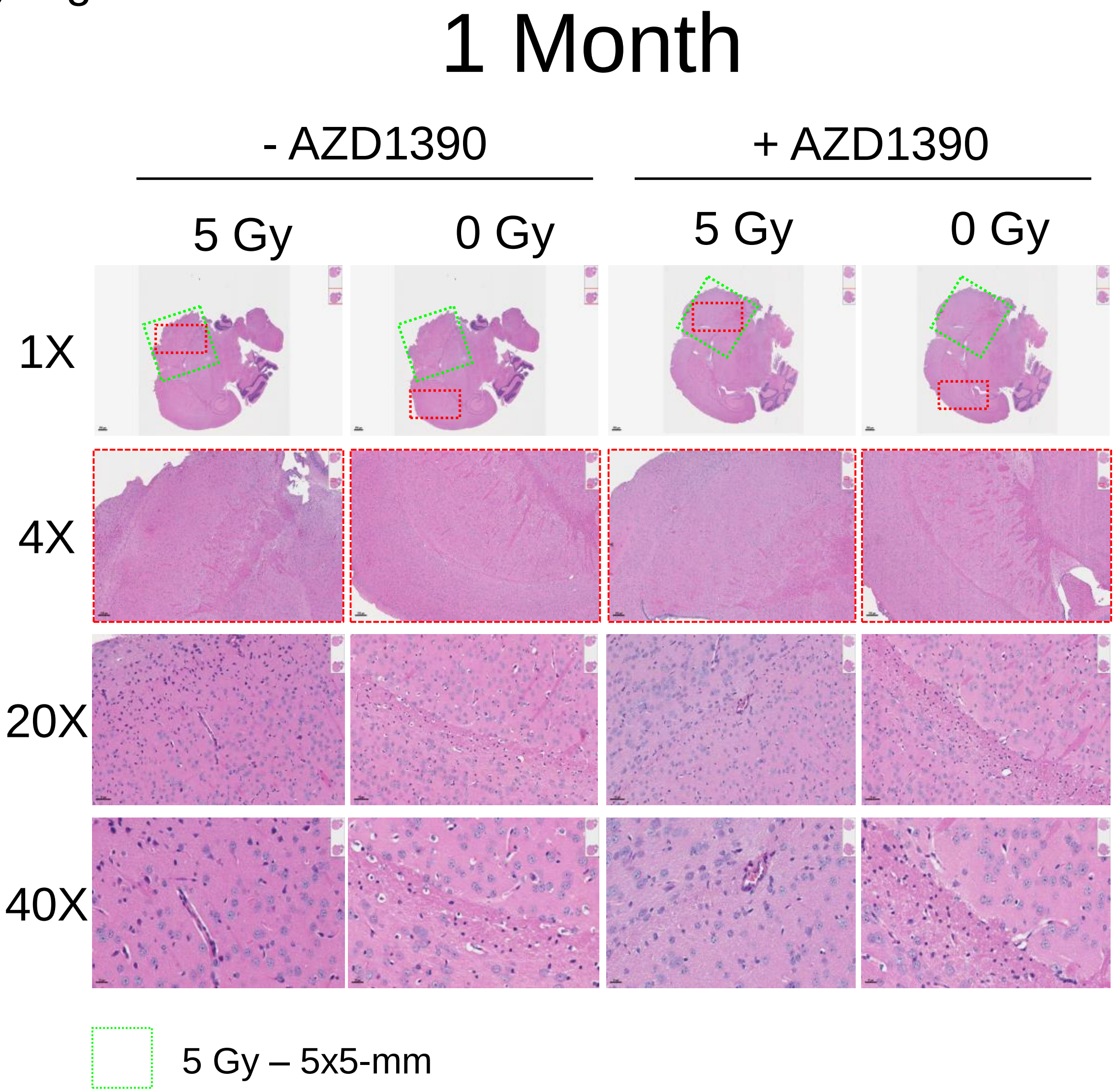

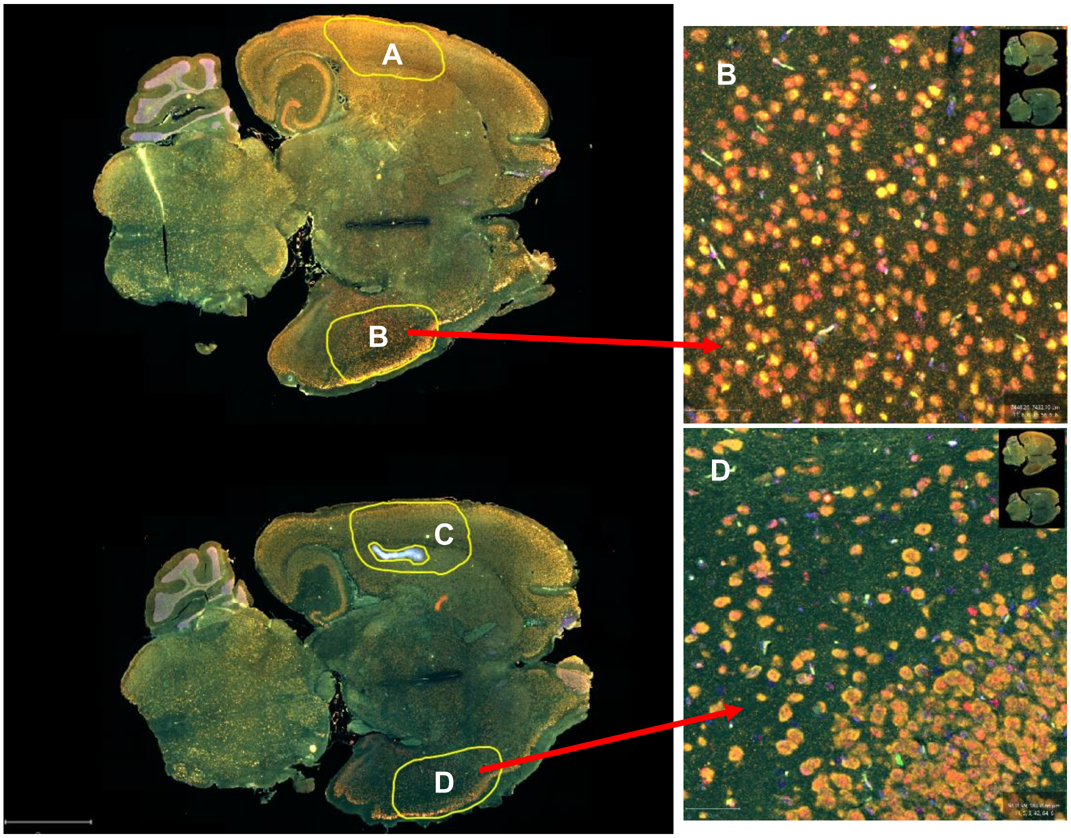

Figure S2. Sections of partially irradiated mice brains that were stained with antibodies and processed for Akoya multiplex immunofluorescence. The samples above were extracted 4 hours after 5 Gy conformal radiation using SARRP. Vehicle (*top section*), AZD1390 (*bottom section*). Area A depicts unirradiated, B depicts irradiated, C depicts AZD1390, and D depicts irradiated after dosing with AZD1390. Antibodies pHH3 (turquoise), PCNA (green); staining control, pKAP-1 (yellow), CC3 (orange), γ-H2AX (red), NeuN (white), and DAPI (blue).

Supplementary Table S1

		Antibody			
**Area/treatment**	**Cell numbers (DAPI)**	**γ-H2AX**	**pHH3**	**CC3**	**CC3: γ-H2AX: pHH3**
Tumor – IR	47203	58	73	107	1
Tumor + IR	51447	8633	9	41	4
NHB - IR	11654	0	7	4	0
NHB + IR	8768	73	12	1	0

Supplementary Table S2

Image	Detections	CC3: PCNA: Neun: pHH3: pKAP	CC3: PCNA: Neun: pHH3	CC3: PCNA: Neun: pKAP1	CC3: PCNA: Neun	CC3: PCNA: pHH3: pKAP	CC3: PCNA: pHH3	CC3: PCNA: pKAP1	CC3: PCNA
Untreated	8480	1	3	0	7	20	33	0	14
IR	9504	0	0	3	9	15	38	2	0
DRUG	9064	1	2	0	4	11	26	0	0
IR + DRUG	9425	0	0	0	0	6	14	0	0

Image	Detections	CC3: Neun: pHH3	CC3: Neun: pKAP1	CC3: Neun	CC3: pHH3: pKAP1	CC3: pHH3	PCNA: Neun: pHH3	PCNA: Neun: pKAP1	PCNA: Neun
Untreated	8480	35	0	447	0	13	23	0	78
IR	9504	5	184	918	1	3	4	3	23
DRUG	9064	34	0	104	0	2	19	0	87
IR + DRUG	9425	5	0	458	0	0	1	0	5

Image	Detections	PCNA: pHH3: pKAP1	PCNA: pHH3	PCNA: pKAP1	Neun: pHH3	Neun: pKAP1	pHH3: pKAP1	H2AX: CC3: PCNA: Neu	γ-H2AX: CC3: PCNA: pHH3: pKAP1
Untreated	8480	4	293	3	165	0	2	0	0
IR	9504	7	249	23	8	89	3	3	0
DRUG	9064	8	588	0	315	1	2	0	1
IR + DRUG	9425	3	268	1	43	1	0	0	0

Image	Detections	γ-H2AX: CC3: PCNA: pKAP1	γ-H2AX: CC3: PCNA	γ-H2AX: CC3: Neun: pKAP	γ-H2AX: CC3: Neun	H2AX: CC3: pHH3: pKAP	γ-H2AX: CC3: pHH3	γ-H2AX: CC3: pKAP1
Untreated	8480	1	0	0	10	0	0	0
IR	9504	0	1	161	108	2	1	53
DRUG	9064	0	0	0	0	0	0	0
IR + DRUG	9425	0	0	16	14	0	3	30

Image	Detections	γ-H2AX: CC3	γ-H2AX: PCNA: Neun: pKAP	γ-H2AX: PCNA: Neun	γ-H2AX: PCNA: pHH3	γ-H2AX: Neun: pKAP1	γ-H2AX: pHH3	γ-H2AX: pKAP1
Untreated	8480	4	0	1	0	0	0	0
IR	9504	20	1	5	1	30	0	24
DRUG	9064	0	0	0	0	0	0	0
IR + DRUG	9425	17	0	0	0	5	2	3
